# A sufficient role of MHC class I molecules on hepatocytes in anti-plasmodial activity of CD8^**+**^ T cells *in vivo*

**DOI:** 10.3389/fmicb.2015.00069

**Published:** 2015-02-12

**Authors:** Jing Huang, Tiffany Tsao, Min Zhang, Urvashi Rai, Moriya Tsuji, Xiangming Li

**Affiliations:** ^1^HIV and Malaria Vaccine Program, Aaron Diamond AIDS Research Center – The Rockefeller UniversityNew York, NY, USA; ^2^Department of Pathology, New York UniversityNew York, NY, USA

**Keywords:** malaria, CD8^+^ T cell, liver, MHC class I, transgenic mouse

## Abstract

Although CD8^+^ T cells are shown to mediate the protective immunity against the liver stages of malaria parasites in mice, whether the direct presentation of malaria antigen by major histocompatibility complex (MHC) class I molecules expressed on the liver of infected host is required for anti-plasmodial activity of CD8^+^ T cells is still unknown. Presently, there is only one CD8^+^ epitope, SYVPSAEQI, derived from the circumsporozoite protein of *Plasmodium yoelii* (PyCS), that mediates anti-malarial protection and is presented in the context of a K^d^ molecule. Therefore, to investigate the mode of anti-plasmodial activity of CD8+ T cells, we have previously generated C57BL/6 transgenic (Tg) mice, in which a K^d^ molecule is expressed only on hepatocyte (Alb-K^d^) or dendritic cell (DC; CD11c-K^d^), by using albumin promoter or CD11c promoter, respectively. We have also generated MHC-I-K^d^ Tg mice, which express the K^d^ molecule under the MHC class I (MHC-I) promoter, as a positive control. From splenocytes collected from CD11c-K^d^ Tg mice immunized with a synthetic peptide, SYVPSAEQI, which corresponds to the CD8^+^ T-cell epitope of PyCS, emulsified in incomplete Freund’s adjuvant , a PyCS-specific CD8^+^ T-cell line was generated. This PyCS-specific CD8^+^T-cell line was then adoptively transferred into a cohort of either MHC-K^d^ Tg or Alb-K^d^ Tg mice listed above, as well as wild-type C57BL/6 mice. Then both transferred and non-transferred mice were challenged with live malaria parasites. We found that the adoptive transfer of a PyCS-specific CD8^+^ T-cell line resulted in a significant inhibition of the parasite burden in the liver of Alb-K^d^ Tg, as well as MHC-I-K^d^ Tg mice, but not of C57BL/6 mice. These results indicate that the K^d^ molecule expressed by hepatocytes is sufficient in mediating the anti-plasmodial activity of PyCS-specific CD8^+^ T cells *in vivo*.

## INTRODUCTION

Malaria is a severe disease that ranks among the most prevalent infections in tropical areas throughout the world. Approximately 250–300 million people become infected yearly with relatively high rates of morbidity and mortality. The WHO estimates that every year nearly one million children die of malaria in Africa alone (World Health Organization [WHO], 2008). The widespread occurrence and the increasing incidence of malaria in many countries, caused by drug resistant parasites and insecticide resistant vectors (*Anopheles mosquitoes*), underscore the need for developing new methods of controlling this disease, which includes more effective vaccines.

A number of mouse studies to date using *Plasmodium yoelii* and *P. berghei* parasites for challenge have shown that protective immunity against pre-erythrocytic stages is mediated in part by T cells, particularly CD8^+^ T cells (Weiss et al., 1988, 1992; Rodrigues et al., 1991, 1997; Tsuji et al., 1998; Sano et al., 2001; Schmidt et al., 2008). Then, the essential role of major histocompatibility complex class I (MHC-I) molecules in mediating CD8^+^ T-cell-dependent anti-malarial immunity was shown by the study in which adoptive transfer of malaria-immune splenocytes into β2 microglobulin (β2 m)-deficient mice failed to confer protection (White et al., 1996). Using transgenic (Tg) mice that express a T-cell receptor (TCR), based on the TCR sequence of CD8^+^ T cells that recognize an immunodominant T-cell epitope of the PyCS protein, SYVPSAEQI, a recent study showed that the Tg T cells do not require bone marrow-derived antigen-presenting cells (APCs) for protection upon adoptive transfer; instead, they recognize antigen on parenchymal cells, presumably parasitized hepatocytes (Chakravarty et al., 2007). These studies all together strongly suggest that MHC-I molecules expressed by malaria-infected hepatocytes play a key role in mediating the anti-plasmodial activity of CD8^+^ T cells *in vivo*. However, it is still unknown to which extent MHC-I molecules expressed by hepatocytes mediate the anti-plasmodial activity of CD8^+^ T cells *in vivo*.

The immunodominant T-cell epitope, SYVPSAEQI, described above is presented by H-2K^d^ (K^d^) molecules to CD8^+^ T cells and is known to be the only epitope that can induce protective CD8^+^ T cells against malaria. Therefore, to provide answers concerning the mechanisms underlying the anti-plasmodial activity of CD8^+^ T cells, we have taken an approach in which Tg C57BL/6 mice, expressing H-2K^d^ molecules only on the surface of hepatocytes (Alb-K^d^), DCs (CD11c-K^d^), or all nucleated cells (MHC-I-K^d^), have been generated (Huang et al., 2013) and used in the current studies.

## MATERIALS AND METHODS

### PARASITES AND ANIMALS

Female *Anopheles stephensi* mosquitoes infected with *P. yoelii* 17XNL strain were purchased from the New York University insectary. *P. yoelii* sporozoites were isolated from the salivary glands of infected *A. stephensi* mosquitoes 14 days after the mosquitoes had received an infectious blood meal (Huang et al., 2014). Six- to eight-week-old C57BL6 mice were purchased from Taconic (Germantown, NY, USA). Three tissues specific H-2K^d^ Tg mice models, Alb-K^d^, CD11c-K^d^, and major histocompatibility complex-I-K^d^ (MHC-I-K^d^) Tg mice models, were established in our lab, in which H-2K^d^ were expressed under the control of albumin, CD11c, and MHC I promoters, respectively, in C57BL/6 mice (Huang et al., 2013, 2014). All mice were maintained under standard conditions in The Laboratory Animal Research Center of The Rockefeller University. Furthermore, all animal experiments were carried out in strict accordance with the Policy on Humane Care and Use of Laboratory Animals of the United States Public Health Service. The protocol was approved by the Institutional Animal Care and Use Committee (IACUC) at The Rockefeller University (Assurance # A3081-01).

### PEPTIDE, TETRAMER, AND CULTURE MEDIUM

A peptide, SYVPSAEQI, which corresponds to the CD8^+^ T cell epitope of the CS protein of *P. yoelii* (PyCS), was synthesized by Peptide 2.0 Inc. (Chantilly, VA, USA). An allophycocyanin (APC)-labeled H-2K^d^/SYVPSAEQI-tetramer was generated and provided to us by the NIH Tetramer Core Facility at Emory University. As culture medium we used DMEM-high glucose (Life Technologies, Grand Island, NY, USA) supplemented with 10% fetal calf serum (Thermo Scientific, Waltham, MA, USA) and 1% supernatant derived from phorbol myristate acetate (Fisher Scientific, Pittsburgh, PA, USA) activated EL-4 cells (Huang et al., 2013).

### GENERATION OF A PyCS-SPECIFIC CD8^+^ T-CELL LINE

CD11c-K^d^ Tg mice were immunized twice with a 2-week interval and intra-peritoneally with10 μg of the synthetic peptide, SYVPSAEQI, emulsified in incomplete Freund’s adjuvant (IFA; Sigma-Aldrich, St. Louis, MO, USA). Ten days after the last immunization, splenocytes collected from immunized CD11c-K^d^ mice were stimulated with radiation-attenuated (3000-rad) splenocytes isolated from naive MHC-I-K^d^ mice pulsed with SYVPSAEQI peptide. We repeated the stimulation for a few times every 10 days, as described Rodrigues et al. (1991), and 10 days after the last stimulation, the frequency of PyCS-specific CD8^+^ T cells among the T-cell line was determined by either Tetramer staining or an ELISpot assay.

### TETRAMER STAINING AND ELISPOT ASSAY

The specificity and frequency of a PyCS-specific CD8^+^ T-cell line was determined by a flow cytometric analysis after staining the T-cell line with antibodies against various CD molecules and an APC-labeled H-2K^d^/SYVPSAEQI-tetramer. All the antibodies used in this study were purchased from BioLegend (San Diego, CA, USA). Briefly, cells were first incubated with unlabeled anti-CD16/CD32 antibody (Clone 16) for 10 min at 4°C. Then the cells were incubated with FITC-labeled anti CD3 antibody (17A2), Pacific Blue-labeled anti-CD8 alpha antibody (clone 53–6.7), and APC-labeled H-2K^d^/SYVPSAEQI-tetramer at room temperature for 30 min. A flow cytometric analysis was performed using an LSR II flow cytometer (BD Biosciences, San Jose, CA, USA). The frequency of SYVPSAEQI-specific, IFN-γ–secreting T cells was determined by an ELISpot assay, as previously described Huang et al. (2013, 2014). Briefly, 1000 cells of the T cell line were co-cultured with 5 × 10^5^ EL-4-K^d^ cells (Huang et al., 2013) loaded with SYVPSAEQI in a well of an ELISpot plate. Twenty-four hours later, the relative number of IFN-γ secreting cells was determined by counting the number of spots that corresponds to IFN-γ-secreting cells using a stereomicroscope. The number of IFN-γ secreting cells after co-culturing with EL-4-K^d^ cells without SYVPSAEQI peptide was used as negative control.

### ADOPTIVE TRANSFER AND PARASITE CHALLENGE

Ten million cells of PyCS-specific CD8^+^ T-cell line were adoptively transferred intravenously (i.v.) into each mouse (Rodrigues et al., 1991). Twenty-four hours later, 1 × 10^4^ viable *P. yoelii* sporozoites were inoculated to each transferred, as well as non-transferred mice by tail vein injection (Rodrigues et al., 1991).

### ASSESSMENT OF PARASITE BURDEN IN THE LIVER

Parasite burden in the liver was determined 42 h after sporozoite challenge by measuring the amount of parasite-specific 18S rRNA in the liver of challenged mice, using a real-time quantitative RT-PCR with the 7500 Fast Real-Time PCR System (Life Technologies, Grand Island, NY, USA; Huang et al., 2014). Parasite burden was described as a ratio of the absolute copy number of parasite-specific 18S rRNA to that of mouse GAPDH.

### STATISTICAL ANALYSES

Statistical analyses were done using GraphPad Prism (version 5.03; GraphPad Software Inc., La Jolla, CA, USA). All data were expressed as the mean ± SD of three mice. Statistical analyses of experimental and control data were evaluated by one-way ANOVA and Student’s *t-*test. A value of *p*<0.05 was considered statistically significant.

## RESULTS

### FREQUENCY OF PyCS-SPECIFIC CD8^+^ T CELLS EXPANDED *IN VITRO* FROM ISOLATED SPLENOCYTES OF CD11c-K^d^ MICE IMMUNIZED WITH SYVPSAEQI PEPTIDE

**FIGURE 1:**
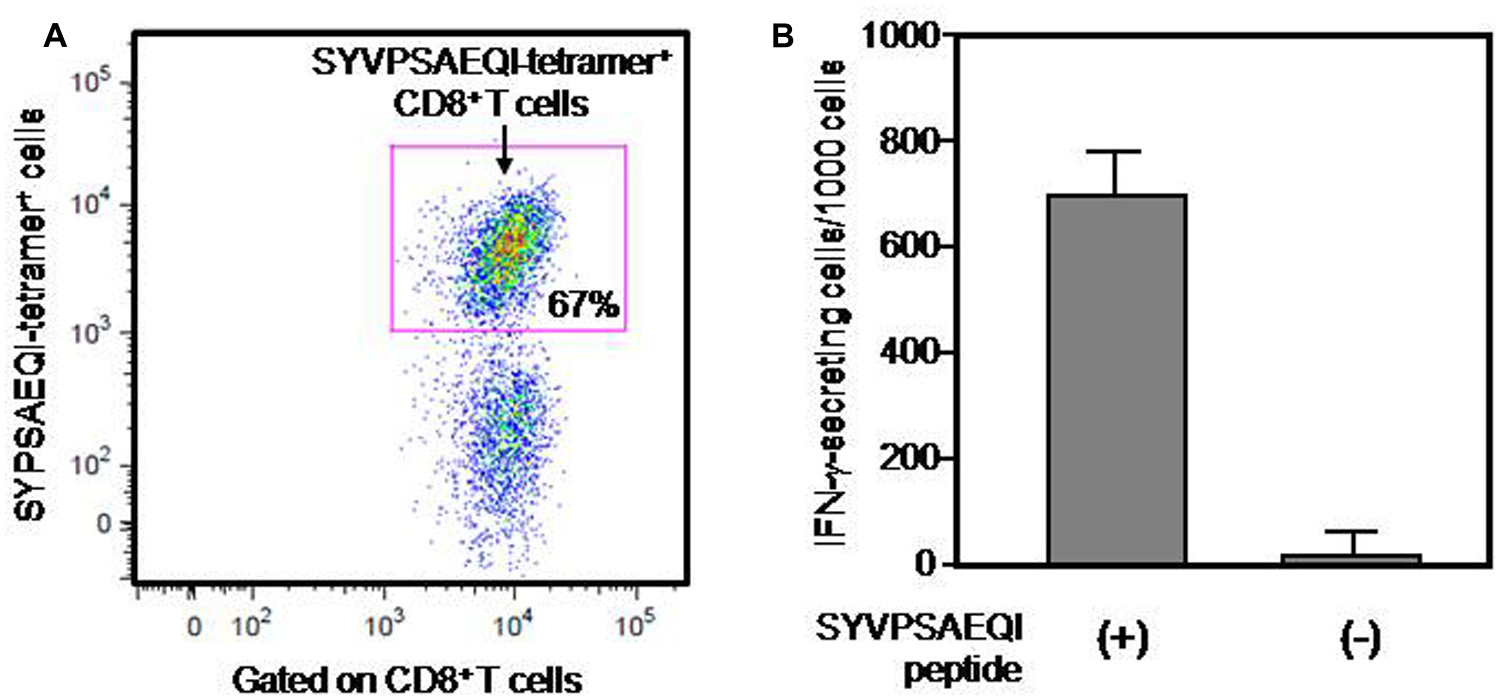
**Frequency of a PyCS-specific CD8^+^ T-cell line, as determined by either a tetramer staining or an ELISpot assay.** Frequency of PyCS-specific CD8^+^ T cells among the T-cell line was determined by either a tetramer staining **(A)** or an IFN-γELISpot assay **(B)**. In **(A)**, CD3^+^ T cells gated with anti-CD8 antibody were stained with APC-labeled H-2K^d^/SYVPSAEQI-tetramer. The number shows the percentage of CD8^+^ T cells that are positive with H-2K^d^/SYVPSAEQI-tetramer. In **(B)**, 1000 cells of the T-cell line were co-cultured with 5 × 10^5^ EL-4-K^d^ cells loaded with SYVPSAEQI in a well of an ELISpot plate, and 24 h later, the relative number of IFN-γ-secreting cells was determined by an ELISpot assay. The number of IFN-γ-secreting cells after co-culturing with EL-4-K^d^ cells without SYVPSAEQI peptide loading was used as a negative control.

CD11c-K^d^ Tg mice were immunized twice with SYVPSAEQI peptide emulsified in IFA with a 2-week interval. Then splenocytes from peptide-immunized CD11c-K^d^ Tg mice were collected for the expansion of a PyCS-specific CD8^+^ T-cell line *in vitro*, which was achieved by stimulating the cells for a few times with irradiated MHC-I-K^d^ Tg mouse-derived splenocytes pulsed with the SYVPSAEQI peptide in a 10–14-day interval, as similarly performed previously (Rodrigues et al., 1991). After successful expansion of a PyCS-specific CD8^+^ T-cell line, the specificity and frequency of the PyCS-specific CD8^+^ T-cell line were determined by either APC-labeled H-2K^d^/SYVPSAEQI-tetramer staining or IFN-γ ELISpot assay, as shown in **Figure 1**. The tetramer staining results depicted that more than two third of the cells of a PyCS-specific CD8^+^ T-cell line could be identified as H-2K^d^/SYVPSAEQI tetramer CD8^+^ T cells (**Figure 1A**). By assessing the ELISpot assay, more than 700 cells out of 1,000 cells of the T cell line were found to get activated by the SYVPSAEQI peptide and secrete IFN-γ, whereas in the absence of the SYVPSAEQI peptide, the cells did not display any responses, indicating that there was no non-specific or auto-reactive T cells present in the T cell line (**Figure 1B**). This indicates that a majority (>65–70%) of the population in the T cell line are specific to the SYVPSAEQI epitope.

### INHIBITION OF *P. yoelii* HEPATIC STAGE DEVELOPMENT IN MHC-K^d^ MICE AND Alb-K^d^ MICE AFTER ADOPTIVELY TRANSFERRING PyCS-SPECIFIC CD8^+^ T CELLS

**FIGURE 2:**
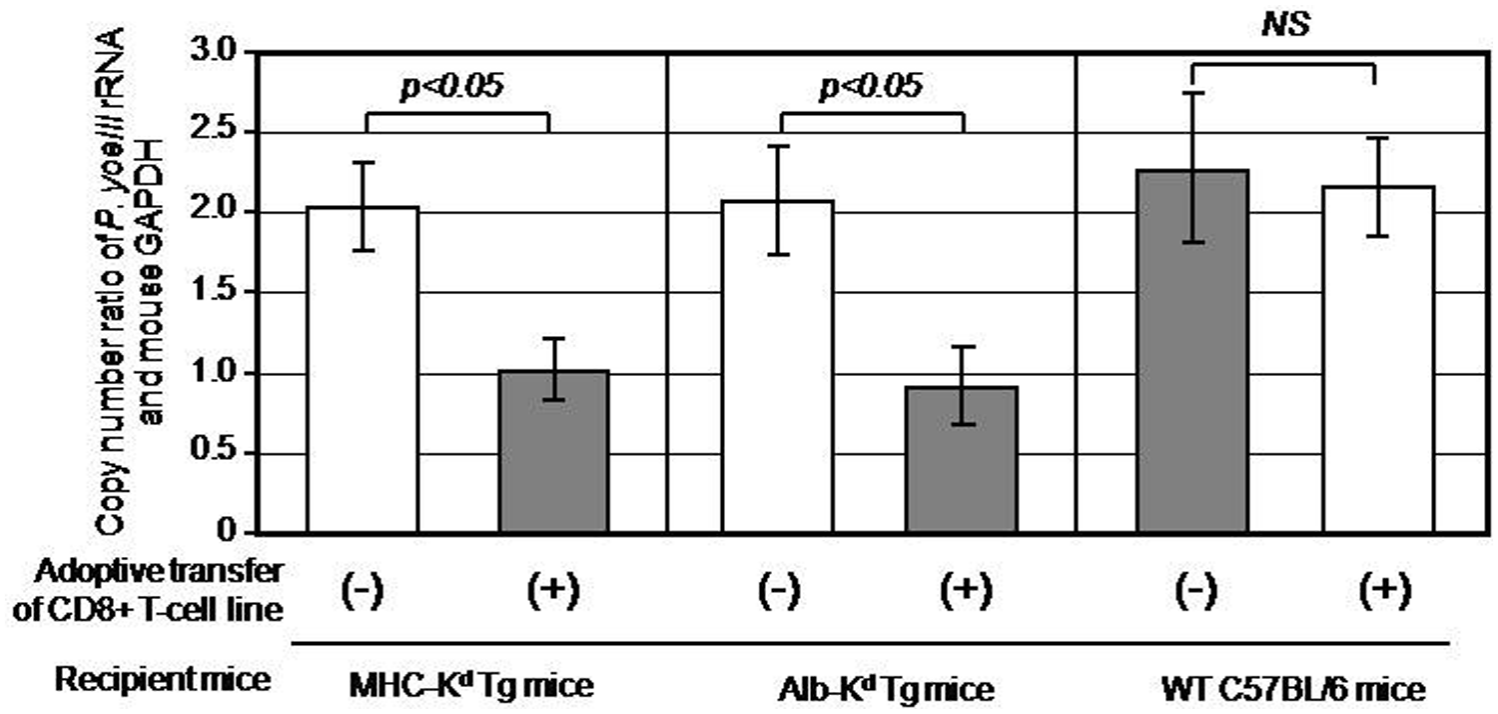
**Inhibition of *Plasmodium yoeli* liver stage development in MHC-K^d^ mice and Alb-K^d^ mice upon adoptive transfer of PyCS-specific CD8^+^ T cells, as determined by a real-time qRT-PCR.** Each mouse of MHC-K^d^ Tg mice, Alb-K^d^ Tg mice, and wild-type C57BL/6J mice (three mice per group) received 1 × 10^7^cells of a PyCS-specific CD8^+^ T-cell line intravenously. Twenty-four hours later, the transferred, as well as non-transferred Tg mice and wild-type C57BL/6 mice, were challenged by 1 × 10^4^ viable *P. yoelii* sporozoites. After 42 h, the parasite burden in the liver was determined by measuring the relative copy number of parasite-specific 18S rRNA to that of mouse GAPDH using a real-time qRT-PCR. The mice that do not receive PyCS-specific CD8^+^ T-cell transfer served as negative controls. Error bars represent means ± SEM (n = 3). A value of p < 0.05 was considered statistically significant, whereas N.S. means “not significant.”

Now that we generated a PyCS-specific CD8^+^ T-cell line having more than two third of the cells specific to SYVPSAEQI peptide, we sought to address one of the key questions regarding the manner in which CD8^+^ T cells recognize and eliminate the hepatic stage of malaria *in vivo*. More specifically, we aimed to determine the role of MHC-I molecules expressed on hepatocytes in mediating CD8^+^ T cells’ recognition of malaria-infected hepatocytes and their anti-plasmodial activity *in vivo*. For this purpose, we adoptively transferred 1 × 10^7^ cells of PyCS-specific CD8^+^ T-cell line to three groups (three mice each): MHC-I-K^d^ Tg mice (as a positive control), Alb-K^d^ Tg mice and wild-type C57BL/6 mice (as a negative control). Respective Tg mice that do not receive the PyCS-specific CD8^+^ T-cell transfer were used as a negative control for each group of transferred Tg mice. All experimental mice were then challenged with 1 × 10^4^ live *P. yoelii* sporozoites. Forty-two hours after the challenge, the livers were collected from challenged mice, and the liver parasite burden was determined by measuring the parasite-specific rRNA by real-time PCR and quantified by a ratio of the absolute copy number of parasite-specific 18S rRNA to that of mouse GAPDH. After the *P. yoelii* sporozoites challenge, the PyCS-specific CD8^+^ T-cell line inhibited almost 50% of the parasite burden in the liver of MHC-I-K^d^ Tg mice, but not in C57BL/6 mice (**Figure 2**). Most importantly, a PyCS-specific CD8^+^ T-cell line transferred to Alb-K^d^ Tg mice could inhibit (55%) the liver stage development as potently as those transferred to MHC-I-K^d^ Tg mice. These results indicate that K^d^ molecules expressed on hepatocytes are sufficient in mediating the anti-parasitic effect of PyCS-specific CD8^+^ T cells *in vivo*.

## DISCUSSION

The role of MHC-I molecules in mediating CD8^+^ T cell-dependent immunity against the liver stages of rodent malaria parasites has been shown by a few studies as described in the introduction section (White et al., 1996; Chakravarty et al., 2007). These studies have clearly led to a conclusion that CD8^+^ T cells are unable to confer protection in the absence of MHC-I molecules (White et al., 1996) and that CD8^+^ T cells do not require bone marrow-derived APCs for protection (Chakravarty et al., 2007). However, whether CD8^+^ T cells need to recognize MHC-I expressed on hepatocytes in order to exert anti-plasmodial effector activity is yet unknown. Therefore, for the purpose of addressing this key question, we have generated B6 Tg mice that express K^d^ molecule only on hepatocyte (Alb-K^d^; Huang et al., 2013) in addition to those that express K^d^ molecules in all nucleated cells (MHC-I-K^d^; Huang et al., 2013) and performed a set of adoptive transfer experiments, in which malaria-specific CD8^+^ T cell-line was transferred to Alb-K^d^ Tg mice, MHC-I-K^d^ Tg mice (as a positive control), and B6 mice (as a negative control).

We first sought to establish a PyCS-specific CD8^+^ T-cell line from MHC-K^d^ Tg mice, which is a rather straightforward approach. However, such T cell-line bears K^d^ molecule, which should cause allogeneic reaction to wild-type C57BL/6 mice (K^d^ negative) upon being adoptively transferred. Therefore, we decided to generate a PyCS-specific CD8^+^ T-cell line from CD11c-Kd Tg mice, in which DCs express K^d^ molecules, and can present SYVPSAEQI peptide to CD8^+^ T cells that lack K^d^ expression.

In the present study, we found that a PyCS-specific CD8^+^ T-cell line adoptively transferred to Alb-K^d^ Tg mice inhibited more than 50% of the liver stage development, which is equal to the degree of inhibition by the CD8^+^ T cell-line found in MHC-I-K^d^ Tg mice. These results indicate that K^d^ molecules expressed on hepatocytes play a major role in mediating the effector phase of anti-malaria CD8^+^ T-cell response *in vivo*.

As we previously shown, we have also generated C57BL/6 Tg mice that express K^d^ only on DCs (CD11c-K^d^ Tg) and macrophages (hCD68-K^d^ Tg; Huang et al., 2013) for the purpose of determining the role of cells other than hepatocytes, like Kupffer cells and DCs, in triggering PyCS-specific CD8^+^ T cells. However, the difficulty of expanding and generating a large number of PyCS-specific CD8^+^ T cells from CD11c-K^d^ Tg or hCD68-K^d^ Tg mice, thus far, has hampered us from determining the role of DCs and Kupffer cells in mediating anti-plasmodial activity of the CD8^+^ T cells. In order to generate a PyCS-specific CD8^+^ T-cell line from these K^d^ Tg mice, we had tried different prime–boost immunization regimens, i.e., a combination of immunizations with a recombinant adenovirus expressing PyCS, a whole irradiated PySpz in addition to SYVPSAEQI peptide, unsuccessfully. It was also documented in our previous research that CD11c-K^d^ Tg or CD68-K^d^ Tg mice elicited much fewer number of PyCS-specific CD8 T cells *in vivo* than that of MHC-I-K^d^ mice after immunization with either SYVPSAEQI peptide or recombinant adenovirus expressing PyCS (Huang et al., 2013). The maximum number of PyCS-specific CD8^+^ T cells we could obtain from a group of three immunized K^d^ Tg mice was 1 × 10^7^ total cells after stimulating them 3–4 times *in vitro* with radiation-attenuated, APCs derived from MHC-I K^d^ Tg mice. The difficulty of expanding a PyCS-specific CD8^+^ T-cell line *in vitro* may be due to the lack of K^d^ expression by CD8^+^ T cells themselves for presenting the peptide to each other. In any case, we are currently attempting to breed and increase to a large number of CD11c-K^d^ Tg or hCD68-K^d^ Tg mice, so that we will be able to expand PyCS-specific CD8^+^ T cells *in vivo* before direct cell sorting in the future.

## Conflict of Interest Statement

The authors declare that the research was conducted in the absence of any commercial or financial relationships that could be construed as a potential conflict of interest.

## References

[B1] Chakravarty S. Cockburn I. A. Kuk S. Overstreet M. G. Sacci J. B. Zavala F. (2007). CD8+ T lymphocytes protective against malaria liver stages are primed in skin-draining lymph nodes. *Nat. Med.* 13 1035–1041 10.1038/nm1628 17704784

[B2] Huang J. Li X. Kohno K. Hatano M. Tokuhisa T. Murray P. J. (2013). Generation of tissue-specific H-2Kd transgenic mice for the study of Kd-restricted malaria epitope-specific CD8+ T-cell responses in vivo. *J. Immunol. Methods* 387 254–261 10.1016/j.jim.2012.10.019 23142461

[B3] Huang J. Tsao T. Zhang M. Tsuji M. (2014). Circumsporozoite protein-specific K(d)-restricted CD8+ T cells mediate protective antimalaria immunity in sporozoite-immunized MHC-I-K(d) transgenic mice. *Mediators Inflamm.* 2014 728939 10.1155/2014/728939 PMC4124204 25132735

[B4] Rodrigues E. G. Zavala F. Eichinger D. Wilson J. M. Tsuji M. (1997). Single immunizing dose of recombinant adenovirus efficiently induces CD8+ T cell-mediated protective immunity against malaria. *J. Immunol.* 158 1268–1274. 9013969

[B5] Rodrigues M. M. Cordey A. S. Arreaza G. Corradin G. Romero P. Maryanski J. L. (1991). CD8+ cytolytic T cell clones derived against the *Plasmodium yoelii* circumsporozoite protein protect against malaria. *Int. Immunol.* 3 579–85 10.1093/intimm/3.6.579 1716146

[B6] Sano G. Hafalla J. C. Morrot A. Abe R. Lafaille J. J. Zavala F. (2001). Swift development of protective effector functions in naive CD8(+) T cells against malaria liver stages. *J. Exp. Med.* 194 173–180 10.1084/jem.194.2.173 11457892 PMC2193458

[B7] Schmidt N. W. Podyminogin R. L. Butler N. S. Badovinac V. P. Tucker B. J. Bahjat K. S. (2008). Memory CD8 T cell responses exceeding a large but definable threshold provide long-term immunity to malaria. *Proc. Natl. Acad. Sci. U.S.A.* 105 14017–14022 10.1073/pnas.0805452105 18780790 PMC2544571

[B8] Tsuji M. Bergmann C. C. Takita-Sonoda Y. Murata K. Rodrigues E. G. Nussenzweig R. S. (1998). Recombinant Sindbis viruses expressing a cytotoxic T-lymphocyte epitope of a malaria parasite or of influenza virus elicit protection against the corresponding pathogen in mice. *J. Virol.* 72 6907–6910. 9658144 10.1128/jvi.72.8.6907-6910.1998 PMC109904

[B9] Weiss W. R. Berzofsky J. A. Houghten R. A. Sedegah M. Hollindale M. Hoffman S. L. (1992). A T cell clone directed at the circumsporozoite protein which protects mice against both *Plasmodium yoelii* and *Plasmodium berghei*. *J. Immunol.* 149 2103–2109. 1517574

[B10] Weiss W. R. Sedegah M. Beaudoin R. L. Miller L. H. Good M. F. (1988). CD8+ T cells (cytotoxic/suppressors) are required for protection in mice immunized with malaria sporozoites. *Proc. Natl. Acad. Sci. U.S.A.* 85 573–576 10.1073/pnas.85.2.573 2963334 PMC279593

[B11] White K. L. Snyder H. L. Krzych U. (1996). MHC class I-dependent presentation of exoerythrocytic antigens to CD8+ T lymphocytes is required for protective immunity against *Plasmodium berghei*. *J. Immunol.* 156 3374–3819. 8617963

[B12] World Health Organization [WHO]. (2008). *World Malaria Report 2008*. Fact Sheet No 94 Geneva.

